# HIV-1 infected monozygotic twins: a tale of two outcomes

**DOI:** 10.1186/1471-2148-11-62

**Published:** 2011-03-08

**Authors:** Loubna Tazi, Hiromi Imamichi, Steven Hirschfeld, Julia A Metcalf, Susan Orsega, Marcos Pérez-Losada, David Posada, H Clifford Lane, Keith A Crandall

**Affiliations:** 1 Division of Epidemiology, Human Genetics and Environmental Sciences, University of Texas, Health Science Center at Houston, School of Public Health, Brownsville Regional Campus, Brownsville, TX, USA; 2 Laboratory of Molecular Retrovirology, Clinical Services Program, SAIC-Frederick, Inc., NCI-Frederick, Frederick, MD, USA; 3 Clinical and Molecular Retrovirology Section, Laboratory of Immunoregulation, National Institute of Allergy and Infectious Diseases, National Institutes of Health, Bethesda, MD, USA; 4 Eunice Kennedy Shriver, National Institute of Child Health and Human Development, National Institutes of Health, Bethesda, MD, USA; 5 Collaborative Clinical Research Branch, Division of Clinical Research, National Institute of Allergy and Infectious Diseases, National Institutes of Health, Bethesda, MD, USA; 6 CIBIO, Centro de Investigação em Biodiversidade e Recursos Genéticos, Universidade do Porto, Campus Agrário de Vairão, Vairão, Portugal; 7 Department of Biochemistry, Genetics and Immunology, University of Vigo, Vigo, Spain; 8 Department of Biology, Brigham Young University, Provo, UT, USA

## Abstract

**Background:**

Replicate experiments are often difficult to find in evolutionary biology, as this field is inherently an historical science. However, viruses, bacteria and phages provide opportunities to study evolution in both natural and experimental contexts, due to their accelerated rates of evolution and short generation times. Here we investigate HIV-1 evolution by using a natural model represented by monozygotic twins infected synchronically at birth with an HIV-1 population from a shared blood transfusion source. We explore the evolutionary processes and population dynamics that shape viral diversity of HIV in these monozygotic twins.

**Results:**

Despite the identical host genetic backdrop of monozygotic twins and the identical source and timing of the HIV-1 inoculation, the resulting HIV populations differed in genetic diversity, growth rate, recombination rate, and selection pressure between the two infected twins.

**Conclusions:**

Our study shows that the outcome of evolution is strikingly different between these two "replicates" of viral evolution. Given the identical starting points at infection, our results support the impact of random epigenetic selection in early infection dynamics. Our data also emphasize the need for a better understanding of the impact of host-virus interactions in viral evolution.

## Background

Over the past few decades, evolutionary biology has had an increasing impact on biomedical research [1-3]. Evolutionary theory can address pertinent questions related to the control of infectious diseases and particularly to pathogen virulence [4]. RNA viruses serve as exciting models for testing this theory because of their potential for rapid evolution. For instance, a striking feature of the Human Immunodeficiency Virus 1 (HIV-1) is the rapid population dynamics resulting in a high degree of genetic diversity within and between infected individuals [5,6]. The virus can then capitalize on this genetic diversity to evade a host immune response [7,8]. Nevertheless, host-virus coevolution is also important with respect to disease progression [9,10]. Viral evolution can be strongly shaped through antiviral pressure applied by the host's immune system [11,12].

A few complications in the study of host-virus coevolution are the different genetic backgrounds of the host and the genetic diversity of the infecting viral population. The model of monozygotic twins infected synchronically through exposure to the same viral population, therefore, provides an opportunity to examine the population dynamics of HIV while holding a number of confounding variables constant. Here we exploit HIV-1 as a model system [13] to examine the population genetic processes and epigenetic influence on viral evolution. We collected nucleotide sequence data from the genes Protease (*pro*), Reverse Transcriptase (*rt*), and Envelope (*env*) in each monozygotic twin. Using these data, we estimated relevant population genetic parameters, including recombination rates, genetic diversity, growth rates, and selection pressure to compare between the two host individuals. While these twins have identical genetic backgrounds and were infected with the same source of HIV (a common transfusion source collected from one donor and administered simultaneously to both twins at birth), they have remarkable differences in their clinical courses. Twin A is almost normal in terms of immune system (total average of CD4 T cell counts: 860 cells/μl for twin A; and 319 cells/μl for twin B) and growth, whereas twin B is almost 5 years delayed in terms of size and had many complications including a rare neoplasm.

Recent studies focusing on monozygotic twins are conflicting on whether HIV evolution can be predictable [14,15] or unpredictable [16,17], related to the immunological repertoire recruitment in these hosts. Those studies examined immune responses, viral evolution, and disease outcome in monozygotic twins infected simultaneously with the same virus. They showed that immune selection driven by dominant sequences in each host could contribute to specific pathways of HIV-1 evolution. The same natural model is also investigated here; however we focus on the evolutionary processes acting on HIV-1 evolution in identical twins by characterizing the population genetic parameters, including positive selection in the viral population. If the viral evolution is predictable, we expect to see similar population dynamics in both twins. However, if there is significant epigenetic impact on viral evolution, then we would see differences in the population dynamics and associated population genetic parameters.

## Results and Discussion

**Figure 1:**
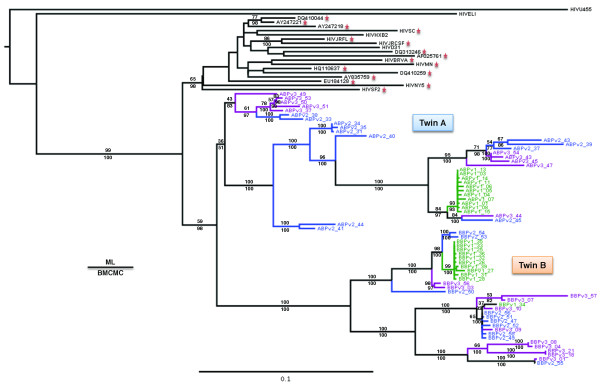
**Midpoint-rooted phylogenetic tree of *env *sequences for the viral populations collected from both twins**. Numbers above and below branches indicate Maximum Likelihood (ML) bootstrap proportions and Bayesian posterior probabilities (as percentages), respectively. Reference sequences (see main text for details) from the US are indicated by a star. Tree branch colors represent three sampled time points in the *env *sequences for twin A and twin B (green: 09/15/1998, blue: 10/13/1998 and purple: 11/10/1998).

**Figure 2:**
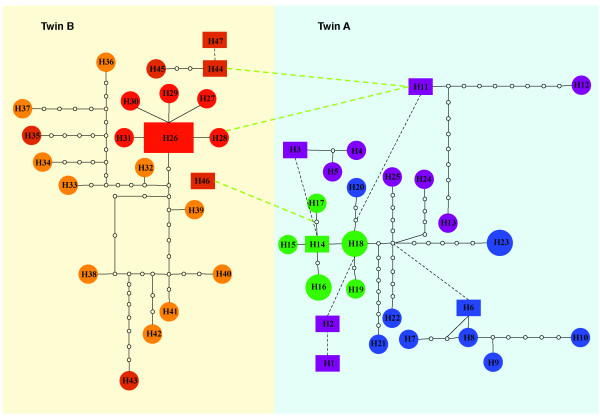
**Statistical parsimony network of *rt *in each twin**. The network is constructed so that the colored squares and circles represent actual cloned sequences. The size of the colored squares and circles is proportional to the number of sequences displaying the same genotype. Each open circle represents putative sequences in the evolutionary pathway. The solid lines on a network represent mutational connections among unique genotypes with at least a 95% degree of confidence, whereas the dashed lines represent a more tenuous connection. Different colors in the network were used to represent three sampled time points in the *rt *sequences for twin A and twin B (green: 09/15/1998, blue: 10/13/1998 and purple: 11/10/1998).

**Figure 3:**
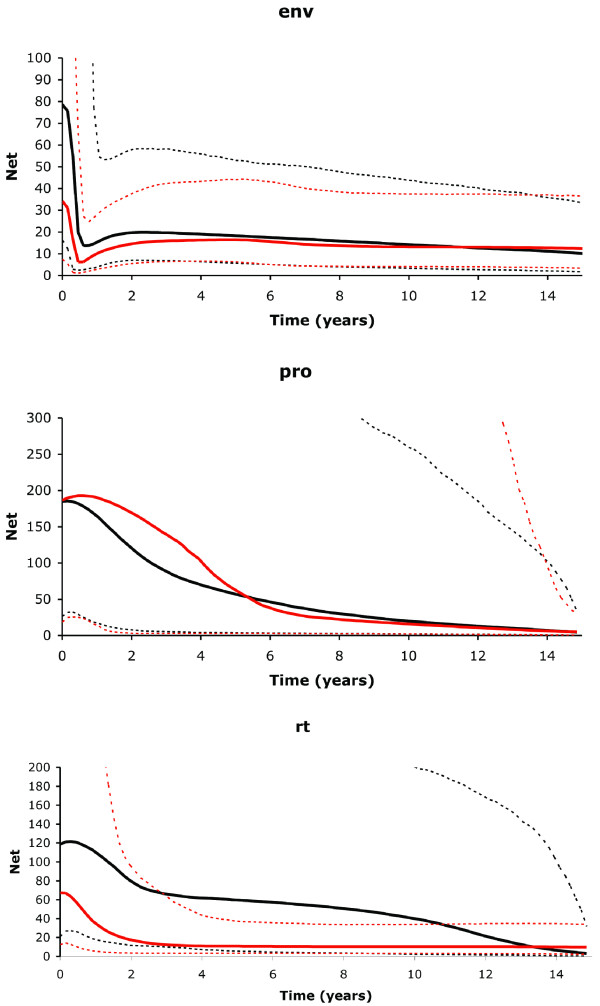
**Bayesian skyline plots of the past population dynamics of HIV-1 in twins A (black lines) and B (red lines) for *env*, *pro *and *rt***. Solid lines show the median estimate and dashed lines the 95% highest posterior density limits.

The phylogenetic analysis of *env *revealed clear evolutionary differences between the viral populations present in each twin (Figure 1), with twin A showing much longer branch lengths compared to twin B. The viral populations from each twin formed reciprocally monophyletic groups with a shared most recent common ancestor compared to HIV-1 control sequences (lab reference sequences and the closest sequences identified in BLAST analyses), as one would expect given the same source population of HIV. Twin A also had much high levels of genetic diversity compared to twin B (Figure 1; Table 1). The phylogenetic network reconstruction of *rt *(Figure 2) also indicates distinct viral populations for each twin, with a relatively higher number of internal sampled genotypes in twin A in comparison with twin B. Twin B has a higher number of tip haplotypes (although not statistically significant with a *P *= 0.10; Fisher Exact Test), suggesting that selection is acting with more force on twin B compared to twin A. Both phylogenetic and network reconstruction analyses showed similar results for all genes analyzed (*env*, *pro *and *rt*) (data not shown). Growth rates were also different between the HIV populations infecting the two twins with twin A showing a rate, at least, two times higher than twin B (Table 1). Interestingly, recombination rates were relatively similar except for *rt *where the recombination rate (*C*) was three times higher in twin A. In both twins the substitution rate was higher than the recombination rate, as indicated by the low estimates of *r *(c/μ). The ratio of the per-site rate of recombination to the per-site rate of mutation (c/μ) in all genes was less than one, indicating that mutation plays a more significant role in producing novel genetic combinations than recombination and is, therefore, the major force driving the evolution of these viral populations [see [18]]. Moreover, the BEAST analyses showed that twin A had a higher relative genetic diversity than twin B and showed different population dynamics through time (Figure 3). In *env*, after a similar starting point in both twins, the relative genetic diversity remained constant until just recently when both twins had a sudden decrease in diversity followed by an exponential increase with twin A increasing to much higher levels of diversity compared to twin B. In *pro*, both twins showed a gradual increase in the relative genetic diversity until just after drug therapy intervention. Then twin B starts to increase in diversity before twin A (perhaps because twin B started drug therapy (AZT) two years prior to twin A (ddI); see methods). Twin A then has a steeper increase in diversity relative to twin B before their levels come together at higher levels. In *rt*, after a short initial starting point in both twins, twin A rapidly develops higher diversity than twin B through time with both patients showing increases in diversity over the last two years, but twin A maintaining a relatively higher level of diversity compared to twin B.

**Table 1:** Estimates of genetic diversity (*θ*), recombination (*r *and *C*), and growth (*g*) for *pro*, *rt *and *env *in each twin.

	Genes	*N_S_*	*θ*	*r *(c/μ)	*C*	*g*
Twin A	*pro*	31	0.44 [0.23 - 0.92]	0.07 [0.02 - 0.17]	0.03 [0.005 - 0.16]	324 [222 - 426]
	*rt*	28	0.26 [0.14 - 0.6]	0.23 [0.1 - 0.38]	0.06 [0.01 - 0.23]	177 [86 - 277]
	*env*	33	0.2 [0.13 - 0.33]	0.03 [0.01 - 0.07]	0.01 [0.001 - 0.02]	42 [19 - 66]

Twin B	*pro*	28	0.2 [0.09 - 0.71]	0.1 [0.003 - 0.47]	0.02 [0.0003 - 0.33]	175 [65 - 480]
	*rt*	25	0.12 [0.07 - 0.24]	0.14 [0.04 - 0.25]	0.02 [0.003 - 0.06]	81 [27 - 165]
	*env*	32	0.1 [0.06 - 0.16]	0.02 [0.01 - 0.07]	0.002 [0.001 - 0.01]	13 [(-18) - 41]

LAMARC's confidence intervals (5% and 95%) around the estimates of each parameter are given between brackets. *N_S _*= Number of sequences.

We also investigated the extent to which natural selection has impacted the viral populations for each twin. Significant evidence of adaptive selection was detected in *rt *from twin B (presumably associated with drug resistance) and in *env *from twin A (presumably associated with immune avoidance) using PAML (Table 2). The Bayesian approach identified 11 positively selected sites (p*P *> 0.95) under model M2 and 13 positively selected sites under model M8 in *env *from twin A. All of the 11 sites detected under model M2 were also found by model M8. Models M2 and M8 also detected one site under selection in *rt *from twin B (S162D). This site is not documented as a drug resistance mutation site in *rt*. Even though they are not detected as positively selected sites in *rt*, we found, in both twins, M184V mutation that is associated with conferring resistance to 3TC. Also we detected T215F and K219Q mutations in *rt *in twin B, which are associated with conferring resistance to AZT. There are 26 positions known to be associated with protease inhibitors [19]. Some amino acid changes were seen in *pro *in both twins, but none of these changes are known to confer drug resistance. A recent study by Nozawa *et al*. [20] pointed out the low sensitivity of PAML for detecting positively selected sites, however this claim has been rejected by Yang *et al*. [21] who provide strong support to the sensitivity of this statistical method for inferring positive selection in DNA sequences and for comparative analysis of genomic data [22]. Recombination can confound the inference of selection. We, therefore, tested for recombination using GARD (Genetic Algorithm for Recombination Detection) that detected a single recombination breakpoint in *env *for both twins and in *rt *for twin B. The REL (Random Effect Likelihood) selection analyses, that took into account the presence of recombination inferred through GARD, clearly indicated that in *env*, positive selection was stronger for twin A, while no difference was detected in the other genes (Table 3).

**Table 2:** Log-likelihood values and parameter estimates (ω, p, and n) for *pro*, *rt *and *env *in each twin.

	Genes	LnL_M1_	LnL_M2_	ω_M2_	p_M2_	PSS_M2_	LnL_M7_	LnL_M8_	ω_M8_	p_M8_	PSS_M8_
Twin A	*pro*	-634.3	-634.3	1	0	0	-634.3	-634.2	1	0	0
	*rt*	-1098	-1098	1	0	0	-1097.9	-1097.9	1	0	0
	*env*	-3552.5	-3526.5	3.6	0.19	11	-3553.4	-3526.6	3.3	0.25	13

Twin B	*pro*	-666.7	-666.7	1	0	0	-666.7	-666.7	1	0	0
	*rt*	-1444	-1441.8	5.8	0.02	1	-1444.1	-1441.7	5.4	0.02	1
	*env*	-3040.5	-3039.4	1	0	0	-3041	-3039.4	1	0	0

LnL = Likelihood values; ω = estimate of *dN*/*dS *(ω_M2 _and ω_M8_); p = proportion of sites under diversifying selection (p_M2 _and p_M8_); PSS = number of positively selected sites with a posterior probability > 0.95 (PSS_M2 _and PSS_M8_). All comparisons between nested models (M1 vs M2, and M7 vs M8) were significant.

**Table 3:** Selection analyses for *pro*, *rt *and *env *in each twin.

		Twin A			Twin B	
	**GARD**	ω*	PSS*	**GARD**	ω*	PSS*

*env*	237	1.19	20	650	0.54	0

*pro*	--	0.31	0	--	0.30	0

*rt*	--	0.24	0	317	0.36	0

The column GARD indicates the location of breakpoints, ω is the estimate of *dN*/*dS*. PSS are positively selected sites for the REL analysis.

*If recombination was detected, the selection analyses were performed taking into account the resulting partition.

Our goal was to investigate HIV-1 evolution in identical twins infected synchronically at birth with the same blood transfusion. We found compelling distinctions between the viral populations from each twin with respect to their population dynamics, phylogenetic structure, growth rates, recombination rates, genetic diversity, and selection pressures. These results were unexpected due to a combination of having identical starting points with respect to both the infecting viral population and the host genetic background. That coupled with the seemingly limited pathways of evolution for both immune evasion and evolution of drug resistance [14,23] would lead one to predict similar patterns of genetic diversity and dynamics in viral populations resulting in similar clinical outcomes. Instead, we found higher growth rates, higher genetic diversity, and higher recombination in *rt *in the healthier twin A compared to twin B. We also found sites under diversifying selection in *env *in twin A whereas twin B had only one site under selection in *rt *(in PAML analysis). Thus, the higher genetic diversity and higher number of selected sites in *env *appear to be associated with slower disease progression, results concordant with that found in a broad study of disease progression in infants [24]. Similarly, the twins differed in their population dynamics and these differed by gene region. The *rt *and *env *regions showed the viral population in the healthier twin A with higher levels of genetic diversity throughout the history of infection even when there were significant shifts in overall levels of diversity. On the other hand, *pro *showed the viral population in twin B with a gradual increase in diversity post drug therapy with a more rapid increase in twin A that was delayed by the same time period as the delay in the RT inhibitor (2 years). This result suggests that the shape of the response to drug therapy in terms of the HIV population diversity might be diagnostic of future disease progression, but further study with larger sample sizes are needed to better test this response as predictive of disease progression. Nevertheless, these twins clearly show very different responses to infection.

This difference in viral population dynamics is concordant with the differences observed in the clinical courses in each twin. The immune system in twin A shows CD4 T cells at an almost normal rate. The immune system in twin B is depressed, hence no strong selective pressure is acting upon its virus population to evolve fast [24]. All these results combined provide strong evidence that, at least in this case, the replicate evolutionary experiment did not result in an identical outcome demonstrating the importance of selective response to random epigenetic factors impacting disease progression [25].

Indeed, some studies in monozygotic twins revealed increasing epigenetic differences with age [25,26]. Additionally, there is a clear potential for founding effects upon infection [27], even in the context of a blood transfusion as the viral population in a shared blood donation is certainly reduced in genetic diversity and number compared to infectious virus from an infected individual. The combination of a reduced effective population size coupled with strong selective pressure is a key ingredient for founder effects [28,29], resulting in populations with very different characteristics as evident here in both the population dynamics and immunology. Clearly, the early impact of founder effects and epigenetic factors on viral population dynamics has diversifying impact over time as the viral populations undergo independent evolution - even in the face of similar genetic selection pressures, identical genetic starting points, and identical host genetic backgrounds (Figure 1). This epigenetic drift during development can be either stochastic (especially when impacted by genetic drift) or determined by environmental factors [30]. Host-virus interactions in early stages HIV infection are presumed to have a large impact on the disease course and viral evolution [31-34], yet they are exceptionally difficult to study because researchers are typically not able to design experiments to investigate viral dynamics at infection. Our study capitalizes on the infection of monozygotic twins through a common contaminated blood transfusion to demonstrate that even more complicated epigenetic factors need to be taken into account in developing hypotheses associated with genetic diversity, population dynamics, selection pressure and their association with disease progression.

## Conclusions

We used monozygotic twins infected at birth from the same blood transfusion contaminated with HIV-1 to study the association of population genetic and phylogenetic diversity with disease progression and clinical outcomes. We documented that these twins had very different clinical outcomes with twin A being relatively healthy compared to twin B. Associated with this slower disease progression in twin A, we found phylogenetic differences, higher growth rates, and higher genetic diversity in the HIV population and higher recombination rates in *rt*. We also found differences in population dynamics across all three gene regions. These differences suggest that epigenetic factors are important in disease progression and can impact viral genetic population dynamics.

## Methods

### Patient information

Subjects A and B are monozygotic twins. Due to intrapartum blood loss, they simultaneously received transfusion shortly after birth from a common blood transfusion source in 1983. The parents were notified that the twins were infected with HIV when they were 2 years old and the twins were declared HIV positive in the same year (1985). At that time, they were both asymptomatic. No superinfection was reported during the first five years of age of the twins. Also the twins did not receive additional transfusions since their birth. The twins came to NIH when they were 5 years old (1988). Since then, they have been closely monitored at NIH. The twins started receiving nucleoside analog reverse transcriptase inhibitors at ages 5 (Twin B) and 7 (Twin A). In 1988, twin B started AZT therapy because he was sicker than his brother. Twin A started ddI therapy two years later (1990). Twin A is normal in terms of immune system and growth. In contrast, twin B is 5 years delayed in terms of size and he presented a rare neoplasm, more likely due to HIV infection. Blood samples and clinical information were obtained 15 years after their birth at the NIH Clinical Center, Bethesda, MD (1998). The twins subsequently showed extremely different clinical courses. Samples were collected under protocols approved by the institutional review boards at the National Cancer Institute. Written informed consent was obtained from participants. This research complied with all relevant federal guidelines and institutional policies. Permission was obtained from the parents of the patients for utilization of samples and data for research, analysis and publication.

### HIV-1 sequencing

Peripheral blood mononuclear cells (PBMCs) were isolated from blood samples by Ficoll-Hypaque gradient density at 650 *g *for 30 min, and the cell pellets were stored in liquid nitrogen until use. DNA was extracted from PBMCs using the PureGene genomic DNA isolation kit (Gentra Systems, Minneapolis, MN) and prepared at optimal concentration for nested polymerase chain reaction (PCR) method. Nested PCR amplification of the V1-V5 region of envelope coding gene, the protease gene, and a part of reverse transcriptase gene was performed with the Expand High Fidelity PCR System (Roche Applied Science, Indianapolis, IN) in a 50 μ l reaction containing 1× Expand High Fidelity buffer 3, 200 μ M dNTPs, 2 mM MgCl_2_, 400 nM primers, and 1.75 U of Expand High Fidelity PCR System enzyme mix. Primer sets used to amplify a 1.1-Kbp fragment, encompassing the *env *V1-V5 region were: +6559 (sense) 5'-GGGATCAAAGCCTAAAGCCA-3' and -7648 (antisense) 5'- ACTTCTCCAATTGTCCCTCA-3' in a first round reaction; +6586 (sense) 5'- AATTAACCCCACTCTGTGTTA-3' and -7627 (antisense) 5'- TATCTCCTCCTCCAGGTCTGA-3' in a second round reaction. Amplification of the HIV-1 *pro *was done with the following primer sets: +2165 (sense) 5'- CAGAAGAGAGCTTCAGGTTTGGG-3' and -2588 (antisense) 5'- ACTTTTGGGCCATCCATTCCTGGY-3' in a first round reaction; +2208 (sense) 5'-TCAGAAGCAGGAGCCGATAGAC-3' and -2550 (antisense) 5'-TGGTACAGTCTCAATAGGACTAATGGG-3' in a second round reaction. The part of *rt *was amplified with: +1882 (sense) 5'-GAAGCAATGAGCCAAGTAACAAAT-3' and -3544 (antisense) 5'-GATATGTCCATTGGCCTTGCCCCT-3' in a first round reaction; +1966 (sense) 5'-TTCAATTGTGGCAAAGAAGGGCAC-3' and -3501 (antisense) 5'-TAAGTCTTTTGATGGGTCATAATA-3' in a second round reaction. Each round of PCR consisted of 25 cycles, with the initial denaturation at 94°C for 2 min, followed by 25 cycles of denaturation at 94°C for 15 s, annealing at 50°C for 30 s, and extension at 72°C for 1 min, with the final extension at 72°C for 7 min. The PCR products were purified with the QIA quick PCR purification kit (QIAGEN, Valencia, CA), and then cloned into pCR2.1-TOPO vector using the TOPO TA Cloning Kit (Invitrogen, Carlsbad, CA). The clones for each gene were sequenced using the ABI BigDye Terminator v3.1 Ready Reaction Cycle Sequencing Kit (Applied Biosystems, Foster City, CA). Sequences were then analyzed on an ABI PRISM 3130 × l Genetic Analyzer (Applied Biosystems, Foster City, CA). GenBank accession numbers for the sequences determined in this study are GQ118464 to GQ118640.

### Phylogenetic analysis and network reconstruction

Sequences from each twin were aligned using MAFFT v5.3 [35] and manually edited using MacClade 4.05 [36]. Phylogenetic trees were estimated using the maximum likelihood approach [37] with nodal support assessed via bootstrapping (1,000 pseudoreplicates) [38], as implemented in PHYML [39]. We also estimated phylogenies using Bayesian methods [40] coupled with Markov Chain Monte Carlo (BMCMC) inference as implemented in MrBayes v3.1.2 [41]. Model selection for these analyses followed the procedure outlined by Posada and Buckley [42] and implemented in ModelTest v3.6 (using PAUP*) [43] under the Akaike Information Criterion (AIC) [44]. Two independent BMCMC analyses were run, each consisting of four MCMC chains (one cold and three heated). Each Markov chain started from a random tree and ran for 2.0 × 10^7 ^cycles, sampling every 1000^th ^generation. In order to confirm that the chains converged and mixed well, we monitored the likelihood scores and compared means and variances of all likelihood parameters from independent runs using Tracer v1.4.1 [45]. The relatedness of the twins' infections was assessed by adding 20 HIV-1 reference sequences to the phylogenetic analysis: 11 lab reference strains (HIVU455, HIVELI, HIVSC, HIVHXB2, HIVJRFL, HIVJRCSF, HIVD31, HIVBRVA, HIVMN, HIVNY5 and HIVSF2) and the nine closest sequence matches (Sequence identity: 85%-95%) identified by BLAST analyses (DQ410259, AF025761, AY247221, DQ313246, AY247218, AY835759, EU184128, HQ110637 and DQ410044). Evolutionary relationships among the sequences in each twin were also assessed using the method of statistical parsimony [46], as implemented in the software package TCS v1.21 [47]. This approach allows for the visualization of evolutionary relationships as a network instead of a bifurcating tree which is often more appropriate for viral populations that recombine.

### Population genetic parameters

Genetic diversity (*θ *= 4*N_e_μ*; where *N_e _*is the effective population size and *μ *is the mutation rate per site), recombination rates (*r *= *c*/*μ*; where *c *is the recombination chance per site), and growth rates (*g*) were estimated for each set of genes using the maximum likelihood coalescent approach implemented in LAMARC v2.0.2 [48]. Three independent runs were performed for each gene in order to assess the reproducibility of the different parameter estimates. Furthermore, an estimate of the diversity generated per recombination (*C*) was obtained by multiplying *r *and *θ *[18].

### BEAST analyses

HIV-1 past population dynamics in each twin was inferred using the Bayesian skyline plot [49] and relaxed clock (lognormal) models [50] in BEAST v1.4.2 [51]. The rate of substitution was calibrated based on the age of infection (15 years). The hyperparameter *m *(number of grouped intervals) was set up to 1/4 of the sequences in each dataset. All the Bayesian MCMC output generated by BEAST was then analyzed in Tracer v1.4.1 [45] to test for convergence and mixing and plot population demographics.

### Selection analyses

The extent of natural selection was inferred by estimating the ratio of nonsynonymous to synonymous substitutions (*ω *= d_N_/d_S_) per site and per gene using the codon-based nested models M1 (neutral)/M2 (selection), and M7 (beta)/M8 (beta and *ω*), as implemented in PAML v4 [22]. Model likelihoods were compared using a Likelihood Ratio Test (LRT) to determine the best-fit model. The Bayes empirical Bayes approach was applied to identify the potential sites under diversifying selection as indicated by a posterior probability (p*P *> 0.95) [52]. In order to confirm the PAML analysis, additional *ω *estimates were also calculated in DataMonkey [53]. Positively and negatively selected sites were identified using the REL approach [54]. Recombination was taken into account by screening for recombination breakpoints using GARD and allowing each recombinant fragment to have its own phylogenetic tree [55].

## Authors' contributions

LT participated in the conception and design of the study, carried out the phylogenetic, haplotype network reconstructions, LAMARC and PAML analyses and drafted the manuscript. HI participated in the conception and design of the study, generated nucleotide sequence data and helped to draft the manuscript. SH supervised the sample collection. JAM and SO helped to collect clinical specimens and medical records. MP-L carried out the BEAST analyses and helped to draft the manuscript. DP carried out the GARD and HYPHY analyses and helped to draft the manuscript. HCL participated in the conception, design and funding of the study, supervised the generation of nucleotide sequence data and helped to draft the manuscript. KAC participated in the conception, design, and funding of the study and helped to draft the manuscript. All authors read, discussed the results and approved the final manuscript.
